# Effect of classroom intervention on student food selection and plate waste: Evidence from a randomized control trial

**DOI:** 10.1371/journal.pone.0226181

**Published:** 2020-01-09

**Authors:** Dmytro Serebrennikov, Bhagyashree Katare, Lisa Kirkham, Sara Schmitt

**Affiliations:** 1 Teagasc Food Research Centre, Ashtown, Dublin, Ireland; 2 Department of Agricultural Economics, Purdue University, West Lafayette, IN, United States of America; 3 Evaluation and Learning Research Center, Purdue University, West Lafayette, IN, United States of America; 4 Human Development and Family Studies, Purdue University, West Lafayette, IN, United States of America; University of New Mexico, UNITED STATES

## Abstract

**Background:**

U.S. children are failing to meet the recommended daily 4 cups of fruits and vegetables. New federal guidelines were implemented for healthier school lunches for the National School Lunch Programs (NSLP). Consequently, students waste large amounts of fruits and vegetables. Several organizations advocate implementation of classroom nutrition education programs as a school nutrition policy.

**Methods:**

We conducted a randomized control trial to evaluate the effectiveness of a classroom nutrition education on food consumption behavior of public elementary school students. Our intervention was designed to improve students’ preferences for fruits and vegetables. We collected data using digital-photography, and estimated the amount of fruits and vegetables selected and wasted using ordinary least squares.

**Results:**

The nutrition education program had no impact on the amount of fruits and vegetables selected by the students in the treatment group. We also find no significant difference in the amount of fruits and vegetables wasted by students in the treatment and control group.

**Conclusion:**

Nutrition education did not change students’ consumption behavior, implying the proposed policy might not be optimal. Inducing a behavioral change in elementary school students is an intricate process and might require more than classroom lessons to change their dietary habits.

## Introduction

Childhood obesity is a major U.S. public health concern. Almost 32% of youth in the country are either overweight or obese [1]. The U.S. population older than 1 year has a diet low in vegetables and fruits and high in saturated fat, sodium, and added sugar [2]. U.S. children across all ages are failing to meet the recommended consumption of 4 cups of fruits and vegetables per day [3]. More than 31 million students are served lunch daily through the National School Lunch Program (NSLP) [4]. In response to deficiencies in children’s food-consumption patterns, new federal guidelines were implemented for healthier school lunches for the NSLP [5]. Per these guidelines, schools are required to provide a serving of fruit and vegetable during lunch, and students are required to take a minimum of one serving of fruit or vegetable on their lunch plate as part of meal reimbursement [6]. Since implementation, several reports have indicated an increase in students choosing fruits and vegetables in their lunches provided by school lunchrooms [7,8]. At the same time, research has found that students participating in the NSLP are wasting a large amount of fruits and vegetables in lunch cafeterias [7, 8, 9]. While the increase in food waste does not necessarily imply lower consumption, it is important to evaluate different school nutrition education programs intended to reduce the amount of food waste in school cafeterias. This is consistent with the USDA’s long-term goal to reduce food waste and loss by 50% by 2030 [10].

The purpose of the present study is to evaluate the effectiveness of a classroom nutrition education program on the food selected and wasted by the elementary school students in school lunchrooms. The American Dietetic Association, the Society for Nutrition Education, and the American School Food Service Association are in support of implementing school and classroom nutrition education programs such as a school nutrition policy [11]. Classroom nutrition education programs have been successful in improving nutrition knowledge among school students [12, 13]. However, the final goal of school and nutrition programs is to improve dietary intake in school children during school lunch. Research has shown little robust evidence to suggest the effectiveness of these school nutrition policies toward improving dietary intake in school children during school lunch [14, 15, 16, 17].

We conducted a randomized controlled experiment involving elementary school students at three public schools in a midwestern state in United States. The main aim of the study was to empirically measure school lunch intervention effects on elementary school students’ fruits and vegetables selection and waste. Most of the studies that measure food selection and waste use self-reported data such as 24-hour food recall workbooks [18] or survey questionnaires [19, 20, 21] as a proxy for actual food consumption. To the best of our knowledge, our study is the first to quantify the amount of food selected and wasted on lunch plates by students in the lunchrooms. We used a well-tested digital photography method to quantify the food selection and food waste data.

School environments can be instrumental in implementing policies and programs to support healthy eating among children. Habits developed during early childhood continue into adulthood [22]. Hence, it is important to motivate elementary school students and encourage development of long-term healthy food choices. Our research contributes to the growing literature on school-based nutrition education interventions in the United States that promote selection and consumption of fruits and vegetables in school lunchrooms.

Research has shown that many multicomponent and multiyear school environment nutrition programs have improved nutrition knowledge and self-reported consumption of fruits and vegetables [12, 21, 23]. They have also shown to be successful in improving consumption of fruits and vegetables in school dining halls [24, 25]. Our study differs from these studies as we collected observational, unbiased data on the food selection and waste of students through digital photography. We are able to quantify the amount of food selected and wasted, and also able to separate the food selected and wasted by different food types.

Auld et al. (1998) used a quasi-experimental, multi-component approach to improve student dietary knowledge and consumption by providing special resource teachers, lessons on food preparation, and resources for community food preparation. Previous literature used complementary activities in other environments such as gardening practices [18], additional lunchroom interventions [24], and school-site wellness committees [25]. To better isolate the effect of various environments and interventions on fruit and vegetable consumption, many studies recruit separate treatment groups for different interventions. For example, Prelip et al. (2012) treated one group with multiple components consisting of traditional nutrition education, while the other treatment group was exposed only to traditional nutrition education. Authors found that neither a multicomponent nor a single component nutrition education intervention was effective in increasing fruit and vegetable consumption. In a different study, McAleese and Rankin (2007) combined nutrition education with gardening for one treatment group, while arranging just nutrition education to the second treatment group. Their results indicated only students with access to both nutrition education and gardening increased their fruit and vegetables intake. This divergence in outcomes in multiple component studies might be due to different data collection procedures. For example, Prelip et al (2012) compiled student questionnaires to source consumption data, while McAleese and Rankin (2007) used self-reported data from recall workbooks.

Our study contributes in two major ways to the literature of school nutrition policy evaluation by estimating the effect of a single component intervention on the quantity of fruits and vegetables wasted by elementary school students. First, most of the previous studies have utilized a non-experimental or a quasi-experimental design, thus limiting the validity and restricting the generalizability of their results. We improved on these studies by conducting a randomized controlled experiment to estimate the average treatment effect of a classroom-based intervention on students’ food selection and food waste. Second, most of the previous literature has utilized self-reported survey or questionnaire data for evaluating the effect of nutrition policies on students’ fruit and vegetable consumption. Our study also utilized the digital photography method to quantify amount of fruits and vegetables selected and wasted by the students. We used digital photographs of student lunch trays taken immediately before and after the students had lunch. We compared the lunch tray images with the actual food weight measurements to estimate the actual weight of both food selected and wasted on trays. These two estimates were used as a basis for estimating the amount of food selected and wasted that formed the two outcome variables for statistical analysis. More details on food consumption measurement are provided in food waste measurement section below.

The remainder of the article is organized as follows. First, we describe our experiment, data collection, digital photography method, and the estimation method. We then provide descriptive statistics and summarize the estimation results for food selected and wasted, followed by a discussion of the results, limitations of the study, and conclusion.

## Materials and methods

### Participants

We received parental consent from 135 students in ten second grade classes, from three public schools in a Midwestern state. Two schools had two classrooms each and one school had six classrooms participating in the study. Parents filled in a consent form and a survey providing their child’s demographic information. There were 5 students for whom the parents did not provide demographic information. Similarly, there were 32 students who either brought lunchbox from home or they were absent from the lunchroom on data collection days. Hence, we could not include these students in our analysis, as we did not have any food selection or waste data for them.

The final sample consisted of 98 students. We randomly assigned five classes to the treatment group and the remaining five classes to the control group. Each school had at least one treatment and one control classroom. We randomized at the classroom level to increase the potential for positive externalities of treatment such as the likelihood of classmates exchanging notes about information taught in class. The treatment group had 62 students and the control group had 36 students. As explained earlier, factors such as unavailability of parental consent and missing data led to a small control group sample size. All the students in the treatment classrooms received the treatment, however only those students with parental consents were included in lunchroom data collection. Institutional review board at Purdue University reviewed and approved the data collection protocol for this study.

### Instruments

The intervention was broadly based on the Health Belief Model [26], emphasizing the importance of educational strategies aimed at improving the student knowledge of perceived benefits related to intake of fruits and vegetables. The intervention involved teachers of treatment classrooms implementing a 6-week, bi-weekly curriculum designed to improve students’ knowledge and preferences for fruits and vegetables. Each lesson was 15–20 minutes long. The teachers in the control classrooms taught regular curriculum without any specific nutrition education. Teachers of treatment classrooms were trained by the co-authors in the activities and lesson plans involved in the curriculum. Teachers of treatment classrooms received teaching material, schedule, manual, and all the supporting material for successful implementation of the curriculum. Control classroom teachers received no such training or material. During summer of 2016, the curriculum and the teaching material were designed by the co-authors and a group of 3^rd^ grade teachers not involved in the intervention. The curriculum was developed based on four existing programs: MyPlate Levels 1 and 2, Two-Bite Club, and Put a Rainbow on Your Plate. Table 1 describes the weekly activities and lessons developed for the curriculum. The developed lessons and activities were aligned to science and health teaching standards to enable teachers to teach curriculum required by the state through the nutrition lessons. There were no other programs or interventions related to nutrition education taught to second grade students at any of the schools.

**Table 1:** Nutrition education curriculum content.

Week	Overarching Question	Activity
1	What does it mean to be healthy? What does it mean to eat healthy?	Students labeled a blank copy of the MyPlate diagram after learning the components of healthy meals during both lessons of this week.
2	Why is it important to eat a variety of foods from all food groups?	The concept of nutrients was introduced, and the book, *Two-Bite Club*, was read.
3	What should I eat less of and why? What can I eat instead?	1. This week was focused on teaching children strategies for replacing sweet or salty snacks with healthy choices. 2. ‘Put a rainbow on my Plate’ framework was introduced, as it was incorporated for the rest of the curriculum.
4	Why should we eat fruits and vegetables?	Children learned the nutritional benefits of orange foods and sampled fruits and vegetables that are orange. Final project introduced.
5 and 6	Why should we eat fruits and vegetables?	The nutritional value and tasting of green and blue/purple foods respectively was emphasized. Final project presentation.

*Fidelity Check*: Table 2 presents the descriptive statistics for the fidelity check. As expected, according to the design and implementation of the treatment, fidelity for the intervention was high. Over the course of 6 weeks, teachers reported delivering the lessons with targeted duration and frequency. They also reported that children were engaged in, and enjoyed, the lessons.

**Table 2:** Descriptive statistics for fidelity check.

Fidelity Variables	Mean
Delivery of two lessons per week, each lesson lasting 15–20 min	2.00
	(0.11)
Implementation of the lessons as designed (4-point likert scale)	3.65
	(0.42)
Student engagement	3.56
	(0.46)
Students enjoying the lessons	3.57
	(0.58)

Standard error in parentheses.

### Procedure

The experiment was conducted in Fall 2016. The intervention spanned for a period of 6 weeks beginning in October and ending in November 2016.

We collected bi-weekly lunchroom data for all students in our sample for two outcome variables: 1) amount of fruits and vegetables selected by the students on their lunch plate and 2) amount of fruits and vegetables wasted by the students. The amount of fruits and vegetables consumed can be elicited by taking the difference between fruits and vegetables selected on the lunch plate and wasted. The key feature of our research design is that the food selection and waste data were collected through photographs of students’ lunch plates. For every data collection, we collected two pictures of each student’s lunch plate. Once before the start of lunch, to collect information on the amount of fruits and vegetables selected on the lunch plate, and once after the end of lunch, to collect information on the leftovers/waste.

We adopted the digital photography method for data collection, as it allowed us to record the type of food items selected and wasted by the students without interfering in the established lunchroom processes. The digital photography method has been validated as a reliable and accurate method for collecting school lunchroom food data as compared to other methods, such as physical weighing [15, 27, 28].

### Food waste measurement

Bi-weekly data collection was conducted for the 6-week intervention period. During these 6 weeks, one of the weeks was fall break, and the schools were closed for partial week. As the data collection during this time was not consistent with the other weeks, we dropped this week from the analysis. Hence, we are using bi-weekly data from 5 weeks when the schools were open for the entire week. The data were collected every Monday and Friday of each week. Two trained research assistants were allocated to each school for data collection. They employed the validated digital photography method [29, 30] to collect food selection and waste data. Each student was assigned a placemat with a unique ID that remained the same throughout the study. Before the beginning of each meal, research assistants arranged the placemats on the lunch tables and directed the students to their seats. Students put their lunch trays on the placemats clearly displaying their IDs. Research assistants used digital cameras to capture images of the lunch trays with the lunch mats before the students started their meals. This captured food selection. After the students were finished with their meals, research assistants captured images of the unconsumed food. This captured the food waste.

The school dining hall managers provided us with the weights of one serving size of all fruits and vegetables (food items) offered in the lunchrooms during that semester. These weights served as reference weights, and we verified them by physically measuring the actual serving size of each food item. Photographs of actual servings were also collected as a reference to compare them with the student lunch data. Fruit and vegetable waste from randomly chosen lunch trays were weighted. These weights, in conjunction with the reference weight, were used to calculate the percentage of fruits and vegetables wasted on each plate. This food waste data, and photographs of actual servings, were used to train graders for plate waste estimation.

Three trained independent graders visually estimated before-lunch and after-lunch photographs using the quarter-based method. According to this method, proportions of waste were quantified on a 5-point scale: zero, quarter, half, three quarters, and one (when all fruits and vegetables are wasted). For a food item to be deemed wasted, at least one quarter of the food item had to be left on the plate. To calculate the amount of fruit and vegetable waste, the derived proportions were multiplied by the weight of one serving of corresponding food item in grams. To obtain the amount of food selected on the plate, we used the reference weights of food items.

We employ intraclass correlation coefficient (ICC) to test the agreement of food waste estimates by three graders [15]. The same team of graders rated each food item; hence, we used the two-way random effect model, ICC (2,1). The results from the intraclass correlation test, available in S1 Table, show the degree of agreement between the graders. The correlation coefficient of 0.97 with a 95% confidence interval of [0.965, 0.974] indicate that the three graders were in agreement with their estimates, and that the quarter-based method is a reliable instrument for extracting food selection and waste data from photographs.

### Data analysis

We begin by calculating the average treatment effect for the nutrition education classes on the food selection and waste behavior of the students using a linear random effects model,

$${\left(Selected\right)}_{dij}=\beta_{0}+\beta_{1}\;Treat_{i}+B_{2}X_{i}+B_{3}Day_{d}+\delta_{j}+e_{ij}+u_{dij}$$ (1)

where *Treat*_*i*_ is a dummy variable, which is equal to 1 if student *i* belongs to the treatment group and 0 otherwise. *X*_*i*_ is a vector of student-level demographic control variables such as age, race, gender, parent’s marital status, and parent’s education. *Day*_*d*_ is day fixed effects to control for common shocks that affect all the students on a given day (e.g., bad tasting food). The random portion of the model includes the class-specific deviation from the grand mean, *δ*_*j*_, and the student-specific deviation from a class’ outcome, *e*_*ij*_, while *u*_*dij*_ is the common error term. We corrected the standard errors for heteroscedasticity and cluster at classroom level to control for within classroom correlation.

**Fig 1:**
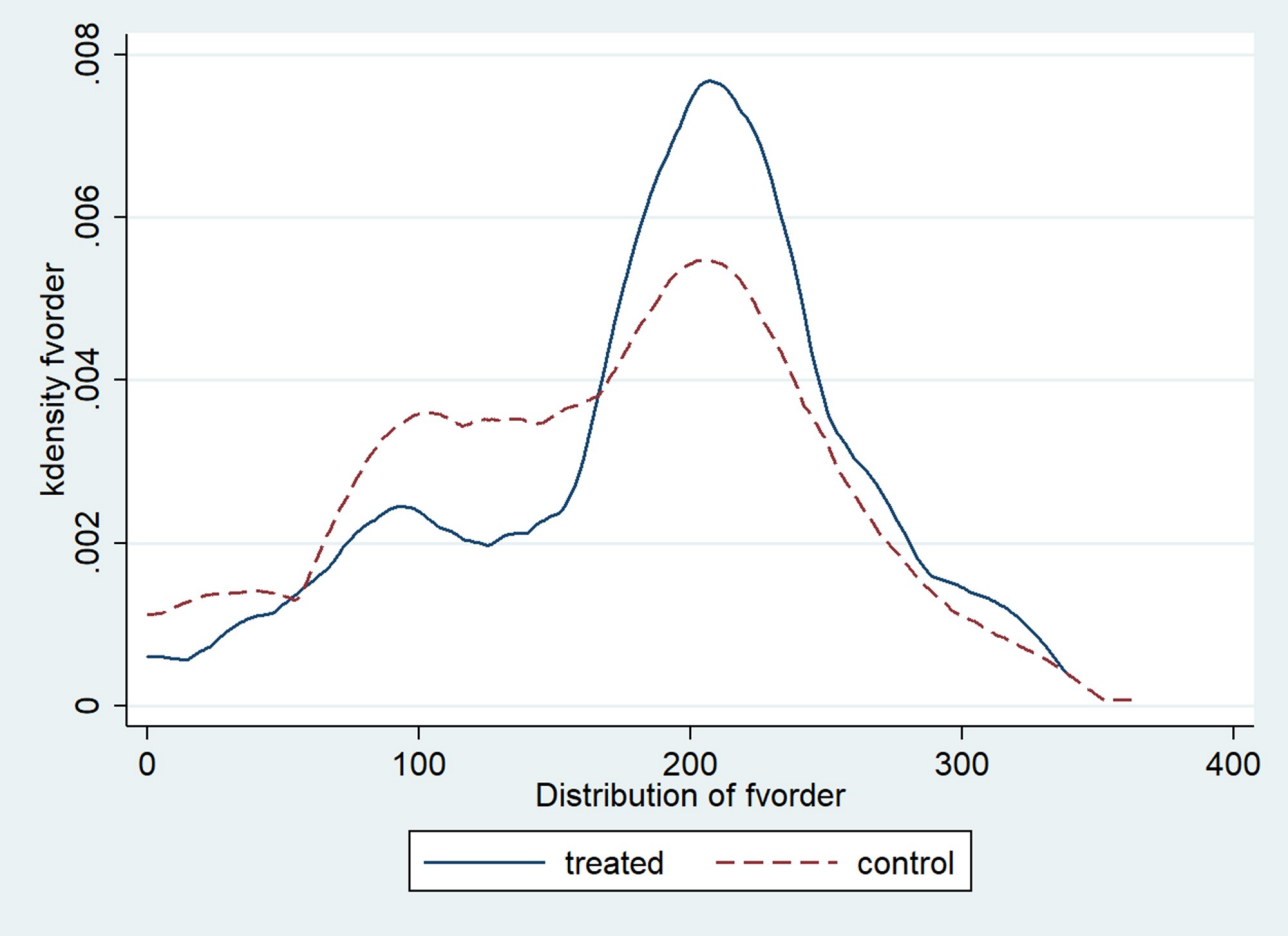
Distribution of fruits and vegetables selected.

**Fig 2:**
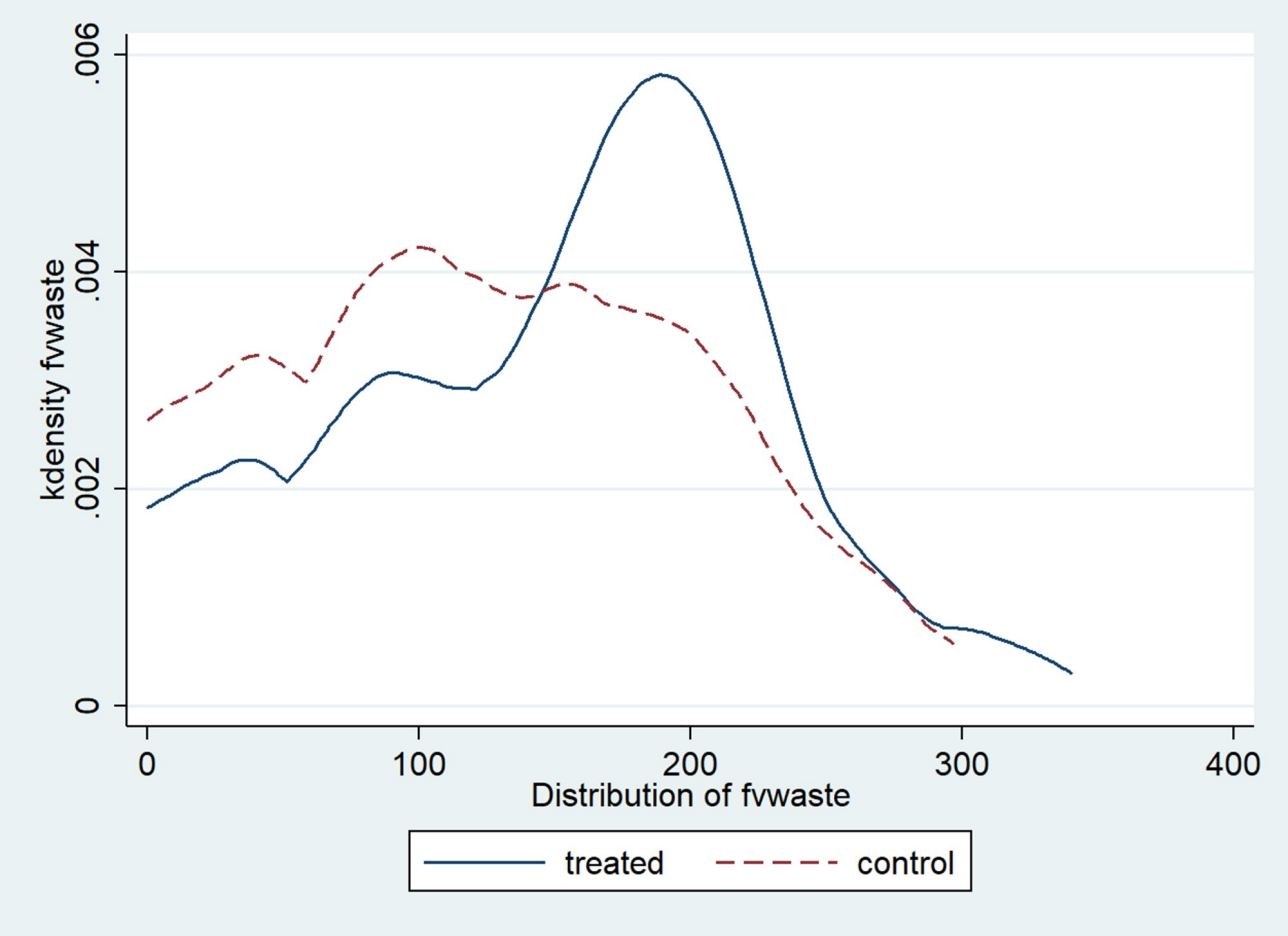
Distribution of fruits and vegetables wasted.

**Fig 3:**
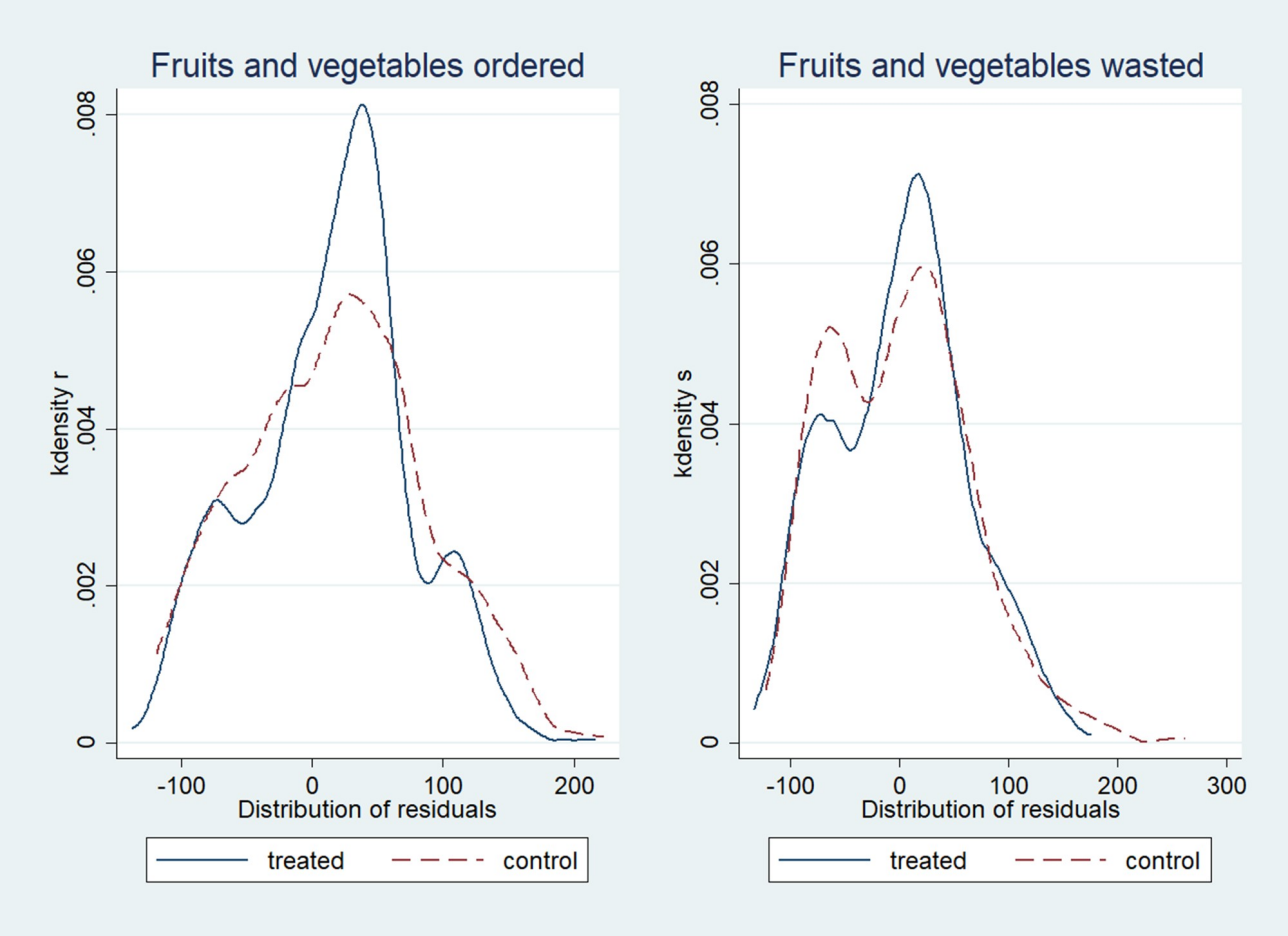
Distribution of residuals from the estimation results for Eq 1.

**Fig 4:**
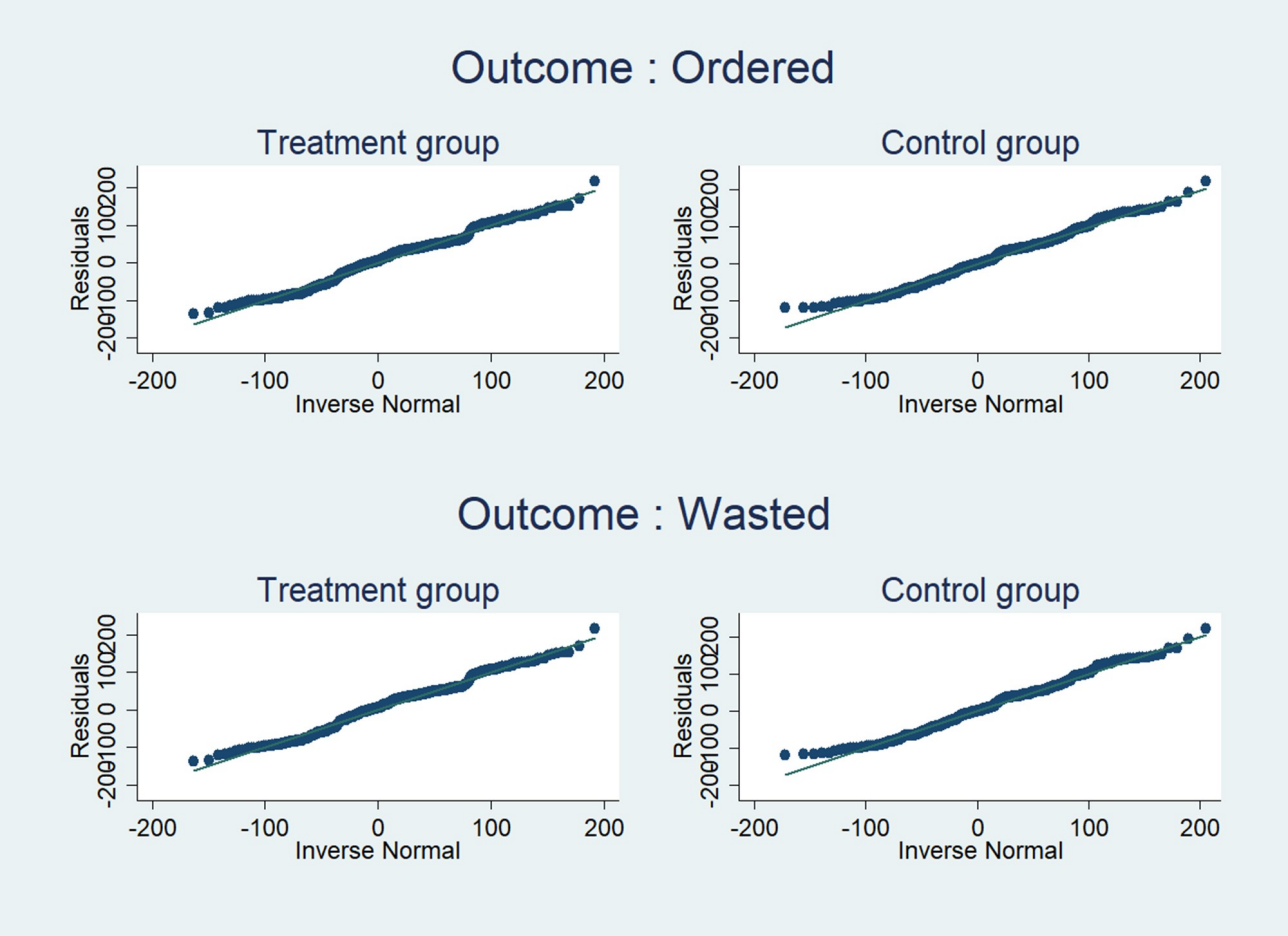
QQ-plots for both outcomes from the estimation results for Eq 1.

We consider two different outcome measures. The first outcome is the amount of food selected on the lunch plate at the beginning of the meal (*Selected*)_*dij*_ by student *i* from class *j* on day *d*. The second outcome is the amount of food wasted at the end of the meal (*Wasted*)_*dij*_ by student *i* from class *j* on day *d*. Figs 1 and 2 show the distribution of the two outcome variables, respectively. The results of diagnostic tests for skewness and kurtosis (S2 Table) show deviation from normality for both variables. Figs 3 and 4 summarize graphical distribution of residuals from model 1. It is shown that the normality assumption generally holds for the residuals from the regression.

As described above, treatment classroom students were taught nutrition education curriculum twice a week for five weeks. To explore any heterogeneity over the treatment time, we interact the treatment indicator with the day indicator. The interaction term between the day and treatment captures the effect of being in the treatment classroom as compared to the control classroom on that day. As mentioned before, this specification contains day fixed effects, and standard errors are corrected for heteroscedasticity and clustered at classroom level to control for within student correlation.

$${\left(Order\right)}_{dij}=\beta_{0}+\beta_{1}\;Treat_{i}+B_{2}(Treat_{i}*Day_{d})+B_{3}X_{i}+B_{4}Day_{d}+\delta_{j}+e_{0ij}+u_{dij}$$ (2)

## Results

The descriptive statistics of students’ baseline characteristics for treatment and control groups is reported in Table 3. The pre-treatment base variables for the sample appear to be well-balanced between treatment and control groups. There is no statistical difference in the pre-treatment baseline variables for the two groups, except for race, with low significance level (p<0.1).

**Table 3:** Mean/frequency comparison of the pre-treatment base variables.

Variable Name	Treatment Group	Control Group	All
Age (months)	95.344	93.757	94.787
	(-11.124)	(-3.791)	(-9.239)
Female	0.442	0.393	0.425
	(-0.5)	(-0.496)	(-0.497)
Parent Marital Status = Married / In Relation	0.77	0.696	0.744
	(-0.424)	(-0.466)	(-0.438)
Parent Education = Bachelor's Degree and higher	0.442	0.515	0.468
	(-0.5)	(-0.507)	(-0.501)
Race = White	0.95	0.848	0.914
	(-0.218)	(-0.364)	(-0.28)
N	62	36	98

Standard error in parentheses.

We also performed a balance test by estimating the pre-treatment student characteristics on a constant and a dummy variable for being in the treatment group. The results for this estimation are reported in Table 4. They suggest that the differences between baseline characteristics for treatment and control group are not statistically significant, as would be expected from random sampling.

**Table 4:** Difference in the base characteristics of students in the treatment and control groups (N = 98).

Variable Name	Difference between Treatment and Control Group
Age (months)	0.932
	(1.128)
Female	0.035
	(0.117)
Parent Marital Status = Married / In Relation	0.046
	(0.111)
Parent Education = Bachelor’s Degree and higher	−0.036
	(0.119)
Race = White	0.107
	(0.073)

Table 5 presents the estimation results for Eq 1 where the outcomes are food selected (column 1) and food wasted (column 2). We see that both food selected and food wasted by the treatment group was higher than the control group during the intervention period. However, the difference is not statistically significant. This implies that the classroom nutrition program had no effect on the food selected and food wasted by the students in the treatment group in school lunchrooms. Noting the direction of the estimates, we see that while treatment group students selected more fruits and vegetables on their plate, they also wasted more fruits and vegetables as compared to the control group. One explanation can be that even if the students were taking more fruits and vegetables on their plates, they were not actively consuming the contents of their plate. This raises the issues of unintended consequences (in this case, food waste) of policies targeted toward improving dietary outcomes. To facilitate the understanding of the effect of the intervention on fruit and vegetable consumption, S5 Table shows the estimates from Eq 1 with fruits and vegetables consumed as dependent variable. As expected, the results do not show any significant effect of classroom nutrition program on consumption.

**Table 5:** Impact of nutrition education intervention on the amount of fruits and vegetables selected and wasted.

Variable Name	Fruits and Vegetables Selected (gm)	Fruits and Vegetables Wasted (gm)
Treatment	25.678	27.729
	(30.766)	(32.881)
Age (months)	-0.241	-0.506
	(0.799)	(0.792)
Female	-22.265	-30.876
	(12.797)	(14.490)
Parent Marital Status = Married/ In Relation	-16.274	-12.319
	(9.399)	(8.769)
Parent’s Education = Bachelor’s Degree and higher	-11.528	-14.228
	(8.048)	(8.076)
Race = White	-3.598	8.785
	(20.499)	(22.008)
Day 1	40.434	3.852
	(40.944)	(31.958)
Day 2	54.248**	25.627
	(21.343)	(22.153)
Day 3	28.822	-5.208
	(26.550)	(31.651)
Day 4	15.36	3.656
	(25.577)	(33.030)
Day 5	11.614	-5.522
	(29.045)	(32.602)
Day 6	45.158	35.240
	(30.565)	(31.720)
Day 7	21.565	-19.034
	(15.869)	(20.978)
Day 8	58.756	20.258
	(35.922)	(25.793)
Day 9	34.573	-8.917
	(29.980)	(41.123)
Day 10	*Base*	*Base*
Constant	179.616**	183.887*
	(88.169)	(98.073)
Random effects	60.270	64.528
Observations	499	499

Standard errors in parentheses are corrected for heteroscedasticity and clustered at classroom level.

* p < 0:10

** p < 0:05

*** p < 0:01.

Previous literature [29] has shown that the nutrition education was successful in influencing fruit consumption but not effective for vegetable consumption. We also analyzed the fruit selection, waste, and consumption as well as vegetable selection, waste, and consumption. The results are available in S6 Table for fruits and S7 Table for vegetables. The estimates show that the intervention did not have any significant effect on fruit or vegetable selection, waste, or consumption. Results show that the treatment group participants wasted more vegetables than the control group by 19.77 grams. However, the statistical significance level is low (p<0.1), hence we do not want to over interpret these results.

Table 6 reports the results from the second specification. These results show the weekly effect of the intervention on the outcome variables over the period of intervention. We hypothesized that with the progress of the intervention, treatment group students will significantly increase their fruit and vegetable selection, and decrease food waste as compared to the control group students. The treatment and day interaction coefficient estimates the difference in the fruits and vegetable selection, or waste, between treatment and control group on that particular day. However, the results in Table 6 do not support our hypothesis. There was no significant difference in the selection of fruits and vegetables, or corresponding food waste, over the weeks between treatment and control groups. The coefficients for the interaction effects for the days are not statistically different than each other. Results comparing the coefficients for each day are listed in S3 Table and S4 Table. We see that the amount of food selected by the treatment group on a specific intervention day has no effect on how much food is subsequently selected by this group. In other words, the nutrition education did not result in a gradual increase in the amount of fruits and vegetables selected by the treatment group through the duration of the intervention. The coefficients for day 4 (week 2) for food selected, and day 4 and day 7 (week 4) for food waste, are statistically significant (p<0.1) but imprecisely estimated. However, these results are not consistent throughout the intervention period and might lead to overinterpretation.

**Table 6:** Heterogeneity in the impact of nutrition education on the amount of fruits and vegetables selected and wasted through the intervention days.

Variable Name	Fruits and Vegetables Selected (gm)	Fruits and Vegetables Wasted (gm)
Treatment	25.705	13.181
	(23.382)	(21.256)
Treatment*Day 1	4.423	-0.935
	(40.161)	(27.163)
Treatment*Day 2	-26.869	18.348
	(36.319)	(46.213)
Treatment*Day 3	-20.781	-23.032
	(22.470)	(36.299)
Treatment*Day 4	8.048	29.316
	(26.973)	(27.844)
Treatment*Day 5	-40.244	-13.265
	(33.950)	(36.818)
Treatment*Day 6	60.964	27.910
	(46.527)	(43.565)
Treatment*Day 7	-39.234	23.786
	(59.396)	(45.829)
Treatment*Day 8	-31.781	-20.977
	(37.827)	(36.985)
Treatment*Day 9	-31.227	11.301
	(21.251)	(41.238)
Age (months)	0.028	-0.451
	(0.304)	(0.429)
Female	-1.222	-10.688
	(8.369)	(7.869)
Parent Marital Status = Married/ In Relation	-10.151	-4.200
	(9.834)	(10.006)
Parent’s Education = Bachelor’s Degree and higher	3.070	0.317
	(3.946)	(5.118)
Race = White	4.792(5.314)	7.261(6.901)
Day 1	-8.208	-18.744
	(40.355)	(32.447)
Day 2	26.309	-6.164
	(22.882)	(12.955)
Day 3	-5.443	-18.747
	(19.820)	(19.959)
Day 4	-24.983	-31.705*
	(20.503)	(17.034)
Day 5	-13.744	-26.086
	(19.417)	(22.426)
Day 6	-35.347	-7.627
	(32.066)	(23.939)
Day 7	-2.535	-60.605*
	(15.561)	(17.042)
Day 8	38.328	14.179
	(35.528)	(27.05)
Day 9	7.305	-38.942
	(32.016)	(36.155)
Day 10	*Base*	*Base*
Constant	217.563***	242.689***
	(37.815)	(45.388)
Random effects	60.048	64.509
Observations	499	499

Standard errors in parentheses are corrected for heteroscedasticity and clustered at classroom level.

* p < 0:10

** p < 0:05

*** p < 0:01. Day 10 is used as a reference day in the model. All estimates are in grams.

### Minimum detectable effects size (MDE)

Results show that the classroom nutrition program potentially had very small effects that were not significantly different from zero. However, we want to investigate the largest effect that might have been detected with the given sample size and duration of the treatment. We use the formula in Eq 3 to calculate the MDE. These are ex-post calculations. MDE is the difference between the amount of fruits and vegetables selected by the students in the control and the treatment group.

We use the pilot data collected before the start of the intervention for this analysis. During the pilot data collection, no nutrition education was provided to the students. We randomly assign placebo treatment and control and estimate Eq 1 using all of the controls as specified earlier for the food selected outcome variable. Standard errors are corrected for heteroscedasticity and clustered at an individual level. We performed 1000 simulations to compute the average variance of distribution of the average treatment effect. This variance was used to calculate the MDE using Eq 3.

$$Minimum\;Detectable\;Effect=(t_{0.05}+t_{0.20})*\surd var(\tilde{\beta })$$ (3)

The results for the minimum detectable effect are calculated with 95% confidence interval and 80% power. With the available sample size dependent on the parental consent and school enrollment, we should be able to detect an effect size as small as 25.6 grams of fruits and vegetables selected by the students. If we substitute the standard error from Table 5 in Eq 3, we detect an effect size of almost 95 grams. As our experiment design had group level randomization, it resulted in fewer degrees of freedom. This might be a potential reason for us having a lower power, and also the reason for the difference between the magnitude of effect from the pre-experiment data and the effect from the estimated parameters.

## Discussion

We conducted a single component intervention to encourage consumption of fruits and vegetables by elementary school students in school lunchrooms. The aim was to familiarize students with fruits and vegetables and, consequently, influence their consumption preferences for these food items. We employ a well-established digital photography method for data collection and quantification to avoid self-reporting bias. Results show that the nutrition education had no effect on selection and waste of fruits and vegetables by treatment group students in school lunchrooms. Our results suggest that the implemented school nutrition policy may not be sufficient to motivate healthy eating habits in elementary school students. To develop those habits, a multi-component intervention program combining in-class nutrition lessons with more experiential activities, like repeated tasting of vegetables or developing cooking skills, might be required. Repeated taste exposure has shown to increase liking of vegetables [31], and cooking programs can positively influence school-aged children’s food preferences [32]. These activities can be implemented to promote hands-on experience in the environment where they can apply the knowledge gained through classroom nutrition education lessons [15]. Another alternative would be to offer a program involving direct financial incentives for taking and eating fruits and vegetables [33].

Previous studies have found mixed results when using various interventions to motivate consumption of fruits and vegetables by students in school lunchrooms. Parmer et al. (2009) posited that nutrition education is not enough to encourage students to consume more fruits and vegetables. Domel et al. (1993) showed that nutrition education in classrooms, targeting both fruit and vegetable consumption, increased preferences for fruits but not for vegetables among elementary school students. Perry et al. (1998) conclude the multicomponent nutrition programs in schools can have positive impact on fruit and vegetable consumption among children, whereas Blom-Hoffman et al. (2004) as well as Prelip et al. (2012) did not find any significant improvement in fruit or vegetable consumption after multicomponent intervention. Using a novel methodology in estimating the effect of nutrition education on consumption of fruits and vegetables, our results complement the results found in previous studies.

According to Amin et al. (2015), after implementation of the NSLP program, food waste in lunchrooms increased by 56%. Our attempt to reduce the amount of fruits and vegetables wasted by employing school nutrition education program had no effect. This short-term, single-component nutrition education program proved ineffective in motivating healthy food consumption habits among elementary school students.

### Limitations

The current experiment design has several shortcomings. The first limitation is a relatively homogenous and small sample size. However, as we collected biweekly data over a period of five weeks, we had enough power to estimate an effect size of as small as 20.10 grams. It will be important for future research to work with larger and diverse student samples. The second limitation is the short duration of the intervention. Changing eating habits, not only in children but also in adults, takes time. Hence, longer intervention period might be required. The third limitation is aggregating the amount of fruits and vegetables at individual level for both selected and wasted. Aggregating the data restricts exploring the possible changes in student’s consumption behavior for individual components. The fidelity check was conducted using self-reported information from the teachers. There is a possibility of overestimation of fidelity as there might be a bias toward reporting higher fidelity by the teachers. The nutrition education lessons were based solely on declarative knowledge that might have restricted children’s adaptation of practical eating habits [34]. Further research is required to study the effect of lessons based on procedural knowledge or a combination on children’s consumption behavior. Another important limitation of our study is that the group randomization resulted in fewer degrees of freedom for our statistical analysis. This might have been a potential reason for a lower statistical power to detect any significant effect.

## Conclusion

Our study is the first to quantify the amount of food selected and wasted by elementary school students in school lunchrooms. The intervention was designed following well-established Health Belief Model. The intervention was not able to motivate a change in student consumption behavior. Our study tests the introduction of classroom nutrition education as a school nutrition policy advocated by different stakeholders. Our findings have implications for school-based interventions that aim to promote healthy eating habits. Our study shows that students are not motivated to take healthy foods on their plates and are on average wasting 76% of the food on their plates. With increasing rate of childhood obesity and diabetes, it is important to test and develop strategies that target school environmental factors that promote healthy dietary habits.

Elementary school students are in an age of forming life-long habits. Policy makers and school officials need to work together to create an environment that would promote and foster healthy eating habits. This needs systematic approach in the form of long-term, multicomponent nutrition education programs. These programs should encompass hands-on activities, interesting to elementary school students, for raising awareness about benefits of consuming fruits and vegetables both at school and at home. Failure to create such stimulating environments may lead to less healthy youth in the forthcoming years and inefficiencies in resource utilization.

## Supporting information

S1 Table (DOCX)

S2 Table (DOCX)

S3 Table (DOCX)

S4 Table (DOCX)

S5 Table (DOCX)

S6 Table (DOCX)

S7 Table (DOCX)
