# Supplementary material for: Effect of classroom intervention on student food selection and plate waste: Evidence from a randomized control trial
Source: PLoS One. 2020 Jan 9;15(1):e0226181. doi: 10.1371/journal.pone.0226181 (PMC6952251; doi:10.1371/journal.pone.0226181)
Supplement: S1 Table — (DOCX) [file pone.0226181.s001.docx]

**S1 Table: Intraclass correlation coefficients (ICC) and their 95% confidence intervals for three graders**

| Rating | ICC | 95% Confidence Interval | |
| --- | --- | --- | --- |
| Individual | 0.970 | 0.965 | 0.974 |
| Average | 0.989 | 0.988 | 0.991 |
