# Supplementary material for: Effect of classroom intervention on student food selection and plate waste: Evidence from a randomized control trial
Source: PLoS One. 2020 Jan 9;15(1):e0226181. doi: 10.1371/journal.pone.0226181 (PMC6952251; doi:10.1371/journal.pone.0226181)
Supplement: S2 Table — (DOCX) [file pone.0226181.s002.docx]

**S2 Table: Skewness/Kurtosis test for normality**

| Variable | Obs | Prob (Skewness) | Prob (Kurtosis) | Adjusted chi^2(2) | Prob>chi2 |
| --- | --- | --- | --- | --- | --- |
| Outcome variables | | | | | |
| Selected (treatment) | 561 | 0.000 | 0.778 | 30.900*** | 0.000 |
| Wasted (treatment) | 561 | 0.025 | 0.000 | 36.250*** | 0.000 |
| Selected (control) | 350 | 0.006 | 0.000 | 19.360*** | 0.000 |
| Wasted (control) | 350 | 0.168 | 0.000 | 33.620*** | 0.000 |
| Regression residuals | | | | | |
| Selected (treatment) | 560 | 0.039 | 0.540 | 4.620* | 0.099 |
| Wasted (treatment) | 560 | 0.162 | 0.003 | 10.060** | 0.007 |
| Selected (control) | 344 | 0.867 | 0.249 | 1.360 | 0.506 |
| Wasted (control) | 344 | 0.000 | 0.162 | 15.860*** | 0.000 |
