# Supplementary material for: Effect of classroom intervention on student food selection and plate waste: Evidence from a randomized control trial
Source: PLoS One. 2020 Jan 9;15(1):e0226181. doi: 10.1371/journal.pone.0226181 (PMC6952251; doi:10.1371/journal.pone.0226181)
Supplement: S3 Table — (DOCX) [file pone.0226181.s003.docx]

**S3 Table:  Wald test for the interaction term between treatment and days for food selected**

|  | Treatment*  Day1 | Treatment*  Day2 | Treatment*  Day3 | Treatment*  Day4 | Treatment*  Day5 | Treatment*  Day6 | Treatment*  Day7 | Treatment*  Day8 |
| --- | --- | --- | --- | --- | --- | --- | --- | --- |
| Treatment*Day2 | 0.24 |  |  |  |  |  |  |  |
| Treatment*Day3 | 0.58 | 0.03 |  |  |  |  |  |  |
| Treatment*Day4 | 0.03 | 1.68 | 4.35** |  |  |  |  |  |
| Treatment*Day5 | 2.08 | 0.08 | 1.85 | 10.27*** |  |  |  |  |
| Treatment*Day6 | 0.04 | 0.46 | 1.10 | 0.35 | 1.89 |  |  |  |
| Treatment*Day7 | 0.30 | 0.09 | 0.15 | 1.06 | 0.00 | 0.41 |  |  |
| Treatment*Day8 | 6.92*** | 0.00 | 0.06 | 1.36 | 0.26 | 0.55 | 0.03 |  |
| Treatment*Day9 | 3.10* | 0.01 | 0.52 | 3.04* | 0.12 | 2.91* | 0.01 | 0.07 |

Chi-squared values are estimated with one degree of freedom. Significance levels: * p < 0:10, ** p < 0:05, *** p < 0:01.
