# Supplementary material for: Effect of classroom intervention on student food selection and plate waste: Evidence from a randomized control trial
Source: PLoS One. 2020 Jan 9;15(1):e0226181. doi: 10.1371/journal.pone.0226181 (PMC6952251; doi:10.1371/journal.pone.0226181)
Supplement: S4 Table — (DOCX) [file pone.0226181.s004.docx]

**SI Table:  Wald test for the interaction term between treatment and days for food wasted**

|  | Treatment*  Day2 | Treatment*  Day3 | Treatment*  Day4 | Treatment*  Day5 | Treatment*  Day6 | Treatment*  Day7 | Treatment*  Day8 | Treatment*  Day9 |
| --- | --- | --- | --- | --- | --- | --- | --- | --- |
| Treatment*Day3 | 0.09 |  |  |  |  |  |  |  |
| Treatment*Day4 | 0.69 | 1.16 |  |  |  |  |  |  |
| Treatment*Day5 | 2.07 | 0.18 | 32.31*** |  |  |  |  |  |
| Treatment*Day6 | 0.33 | 0.56 | 0.51 | 10.85*** |  |  |  |  |
| Treatment*Day7 | 0.19 | 0.36 | 0.14 | 2.74* | 0.02 |  |  |  |
| Treatment*Day8 | 0.18 | 0.45 | 1.49 | 0.03 | 0.73 | 0.57 |  |  |
| Treatment*Day9 | 0.76 | 0.42 | 0.06 | 5.87** | 0.01 | 0.04 | 0.59 |  |
| Treatment*Day10 | 0.25 | 0.01 | 0.75 | 0.31 | 0.51 | 0.32 | 0.03 | 0.82 |

Chi-squared values are estimated with one degree of freedom. Significance levels: * p < 0:10, ** p < 0:05, *** p < 0:01.
