# Supplementary material for: Effect of classroom intervention on student food selection and plate waste: Evidence from a randomized control trial
Source: PLoS One. 2020 Jan 9;15(1):e0226181. doi: 10.1371/journal.pone.0226181 (PMC6952251; doi:10.1371/journal.pone.0226181)
Supplement: S5 Table — (DOCX) [file pone.0226181.s005.docx]

**S5 Table: Impact of nutrition education intervention on the amount of fruits and vegetables consumed**

| Variable Name | Fruits and Vegetables Consumed (gm) |
| --- | --- |
| Treatment | -0.344  (15.344) |
| Age (months) | -0.921  (1.126) |
| Female | 22.936*  (10.849) |
| Parent Marital Status = Married/ In Relation | 2.210  (13.378) |
| Parent’s Education Level = Bachelor’s Degree and higher | 10.591  (14.094) |
| Race = White | -21.046  (18.960) |
| Day 1 | 2.101  (5.802) |
| Day 2 | 11.070  (9.089) |
| Day 3 | 4.479  (10.737) |
| Day 4 | -1.384  (13.089) |
| Day 5 | -16.145  (6.972) |
| Day 6 | 2.246  (10.526) |

*Continued*

**S5 Table 5** continued

| Variable Name | Fruits Consumed (gm) |
| --- | --- |
| Day 7 | -5.096  (9.220) |
| Day 8 | -0.514  (11.302) |
| Day 9 | 1.106  (11.060) |
| Day 10 | *Base* |
| Constant | 155.644  (94.090) |
| Random Effect (Class) | 54.310 |
| Observations | 430 |

Standard errors in parentheses are corrected for heteroscedasticity and clustered at classroom level. * p < 0:10, ** p < 0:05, *** p < 0:01. All estimates are in grams.
