# Supplementary material for: Effect of classroom intervention on student food selection and plate waste: Evidence from a randomized control trial
Source: PLoS One. 2020 Jan 9;15(1):e0226181. doi: 10.1371/journal.pone.0226181 (PMC6952251; doi:10.1371/journal.pone.0226181)
Supplement: S6 Table — (DOCX) [file pone.0226181.s006.docx]

**S6 Table: Impact of nutrition education intervention on the amount of fruits selected, wasted, and consumed**

| Variable Name | Fruits Selected  (gm) | Fruits Wasted  (gm) | Fruits Consumed  (gm) |
| --- | --- | --- | --- |
| Treatment | 17.109  (12.212) | -16.188  (12.145) | 10.339  (7.539) |
| Age (months) | -0.616  (0.661) | 1.003  (1.125) | -0.814  (0.949) |
| Female | -11.594  (7.061) | -14.749  (8.732) | 15.767**  (7.850) |
| Parent Marital Status = Married/ In Relation | -15.485  (9.846) | 1.338  (11.321) | -7.800  (10.045) |
| Parent’s Education Level = Bachelor’s Degree and higher | 17.339  (14.824) | -7.984  (12.103) | 16.361*  (8.840) |
| Race = White | -7.708  (17.706) | 31.968**  (12.458) | -19.522*  (10.791) |
| Day 1 | 0.017  (5.043) | -8.002  (5.828) | 5.808  (5.538) |
| Day 2 | 1.413  (4.181) | -6.517  (7.703) | 7.404  (6.839) |
| Day 3 | -4.450  (4.467) | -9.167  (9.065) | 7.792  (8.350) |
| Day 4 | -8.993  (9.091) | -1.028  (7.954) | 2.486  (7.933) |
| Day 5 | -5.296  (4.537) | 2.213  (4.188) | -10.348  (6.157) |
| Day 6 | 3.507  (6.788) | -11.384  (7.333) | 6.269  (8.762) |

**S6 Table 6** continued

| Variable Name | Fruits Selected  (gm) | Fruits Wasted  (gm) | Fruits Consumed  (gm) |
| --- | --- | --- | --- |
| Day 7 | -1.988  (4.326) | -2.448  (9.432) | -1.240  (7.790) |
| Day 8 | 0.254  (4.964) | 4.226  (7.363) | -0.594  (8.050) |
| Day 9 | -5.429  (4.282) | -8.859  (8.892) | 4.057  (10.925) |
| Day 10 | *Base* | *Base* | *Base* |
| Constant | 161.529***  (61.811) | 32.610  (102.333) | 122.546  (85.692) |
| Random effects 39.203 45.772 47.496 | | | |
| Observations | 430 | 430 | 430 |

Standard errors in parentheses are corrected for heteroscedasticity and clustered at classroom level. * p < 0:10, ** p < 0:05, *** p < 0:01. All estimates are in grams.
