# Supplementary material for: Effect of classroom intervention on student food selection and plate waste: Evidence from a randomized control trial
Source: PLoS One. 2020 Jan 9;15(1):e0226181. doi: 10.1371/journal.pone.0226181 (PMC6952251; doi:10.1371/journal.pone.0226181)
Supplement: S7 Table — (DOCX) [file pone.0226181.s007.docx]

**S7 Table: Impact of nutrition education intervention on the amount of vegetables selected, wasted, and consumed**

| Variable Name | Vegetables Selected (gm) | Vegetables Wasted (gm) | Vegetables Consumed (gm) |
| --- | --- | --- | --- |
| Treatment | 26.609*  (14.985) | 24.516*  (9.947) | -10.744  (12.176) |
| Age (months) | -1.118  (1.060) | -0.433  (1.133) | -0.107  (0.573) |
| Female | -15.586  (7.980) | -13.805  (9.903) | 7.168  (6.637) |
| Parent Marital Status = Married/ In Relation | -1.451  (8.984) | -2.269  (12.236) | 10.010  (9.485) |
| Parent’s Education Level = Bachelor’s Degree and higher | -0.131  (12.116) | -4.065  (11.919) | -5.770  (8.860) |
| Race = White | -28.666  (11.679) | -13.156  (10.805) | -1.494  (12.673) |
| Day 1 | -0.768  (6.011) | 0.999  (3.241) | -3.706  (6.677) |
| Day 2 | 4.755  (6.292) | 0.754  (5.700) | 3.665  (5.438) |
| Day 3 | -7.541  (3.348) | -2.887  (8.146) | -3.313  (5.006) |
| Day 4 | -11.000  (7.606) | 0.100  (8.882) | -3.870  (9.072) |
| Day 5 | 2.569  (6.310) | 7.846  (7.229) | -5.796  (5.951) |
| Day 6 | 3.514  (10.435) | 0.038  (5.461) | -4.023  (7.446) |

**S7 Table 7** continued

| Variable Name | Vegetables Selected (gm) | Vegetables Wasted (gm) | Vegetables Consumed (gm) |
| --- | --- | --- | --- |
| Day 7 | -0.272  (6.400) | 0.401  (7.082) | -3.856  (7.750) |
| Day 8 | -1.641  (10.339) | -5.066  (8.665) | 0.080  (7.756) |
| Day 9 | -7.516  (4.933) | -4.891  (3.412) | -2.950  (6.883) |
| Day 10 | *Base* | *Base* | *Base* |
| Constant | 204.893**  (96.663) | 120.927  (100.795) | 33.098  (44.691) |
| Random effects 44.809 42.761 31.583 | | | |
| Observations | 430 | 430 | 430 |

Standard errors in parentheses are corrected for heteroscedasticity and clustered at classroom level. * p < 0:10, ** p < 0:05, *** p < 0:
